# Who participates in a computer-tailored physical activity program delivered through the Internet? A comparison of participants' and non-participants' characteristics

**DOI:** 10.1186/1479-5868-4-39

**Published:** 2007-09-19

**Authors:** Heleen Spittaels, Ilse De Bourdeaudhuij

**Affiliations:** 1 Policy Research Centre Sport, Physical Activity and Health, Belgium; 2 Department of Movement and Sports Sciences, Ghent University, Watersportlaan 2, 9000 Ghent, Belgium

## Abstract

**Background:**

Today, more and more health professionals use the Internet to deliver behavioral change interventions, because of its advantage to reach a wide variety of people at low costs. However, little is known about who is interested in and actually participates in such website-delivered programs. Therefore, the purpose of this manuscript was to examine the characteristics of participants and non-participants (parents recruited through schools) in a computer-tailored physical activity intervention delivered through the Internet.

**Methods:**

Data was collected in two ways. First, 5706 brochures with a call to participate in a physical activity program, with as key element a website-delivered tailored physical activity advice, were distributed indirectly (through their ildren) to parents of all pupils in 14 primary and secondary schools in Belgium. Parents were asked to return the reply card mentioning if they wanted to participate or not. Second, characteristics of participating and non-participating parents were collected by distributing 2000 short questionnaires to pupils between 10–18 years of age, in 12 of the 14 schools. Chi-square analysis and binary logistic regressions were used to compare characteristics of those parents who showed interest (i.e. positive response on reply card) or actually participated (completed online assessment) in a website-delivered physical activity intervention with the characteristics of those parents who showed no interest or did not participate.

**Results:**

In total 1730 pupils (87% respondents), completed the short questionnaire concerning their parents' age, occupation (to derive the socio-economic status) and physical activity habits. The results of the binary logistic regression showed that mothers were more likely to show interest (Odds Ratio (OR) = 1.68, p < 0.001) and participate (OR = 2.27, p < 0.005) in the program than fathers. High socioeconomic status (OR = 3.42, p < 0.001) and being employed (OR = 3.03, p < 0.001) were also significant predictors for showing interest but not for participation. Age and physical activity level did neither predict interest nor participation.

**Conclusion:**

Both younger and older adults as well as physically active and inactive people participated in our online computer-tailored physical activity program when recruitment was done through schools. However, other health-education programs are still needed to reach all segments of the population equally.

## Background

Epidemiological evidence shows that physical inactivity is an independent risk factor for developing coronary heart disease, cardiovascular disease and all-cause mortality [1-4]. Further, research also demonstrated that physical inactivity increases the risk for other chronic diseases as diabetes mellitus type 2 [5,6], anxiety and depression [7,8], different types of cancer [9], and osteoporosis [10,11]. Since the majority of the adults in Western countries does not participate in regular physical activities [12,13] many are at risk and therefore, effective interventions promoting an active lifestyle that can reach large population groups at low costs are of great importance [14].

Face-to-face counseling, as used in primary care, seemed to be an effective method for delivering physical activity interventions [15,16], but it is very time-consuming, and only a small proportion of the population can be reached [17]. An alternative strategy is computer-tailoring, which seems to be effective in changing smoking, diet or physical activity behaviour [14,18-23]. Computer-tailored interventions can be used to mimic face-to-face counselling [17] by giving participants immediate personally adapted advice after completing an electronic diagnostic questionnaire. With the rapid development of the Internet, a new opportunity is created to distribute computer-tailored interventions in a cost-effective manner.

Today there are more than one billion Internet users worldwide [24] and this number is still increasing due to a drop in the cost of Internet connections and improved high speed access. The biggest penetration rate is found in North America (69.7%), followed by Oceania (53.5%) and Europe (38.9%) [24]. At the moment the increase of Internet users is the largest in underserved populations, such as the elderly, lower educated persons and women; and consequently the gaps among age, educational attainment and gender are narrowing [25]. Consequently Internet interventions could reach a wide variety of people at once, at any time and location. In the last years, more and more health professionals have started to use the Internet to deliver behavioral change interventions on various topics such as smoking [26,27], diet [28] and physical activity [29,30]. The former studies mainly focused on the acceptability, feasibility and efficacy of health promotion programs via the Internet, but little is known about the actual reach of these programs, more specific: who participates in interactive Internet interventions and who does not?

A critique often heard with regard to health promoting interventions and also to computer-tailored interventions is that it only makes the healthiest people healthier. It seems that participants in these interventions are employed, high educated and have more positive health behaviors compared to the general population [23,31,32]. In physical activity interventions, participants are often more physically active than the general population [23,32]. Further, some studies showed that more women than men participate in health promoting interventions through the Internet [33-36].

A study by McClure [37] showed that characteristics of visitors to a smoking cessation website (Project Quit) differed by the recruitment strategy that was used. Participants recruited by newsletters were more likely to be female, Caucasian and older compared to participants who were recruited by a proactive invitation letter. However, the total sample was similar to participants who enrolled in phone counseling smoking cessation programs: participants were middle-aged and moderate-to heavy smokers with a history of numerous quit attempts. Further, Cobb and Graham [38] determined the characteristics of adults who search the Internet for smoking cessation information. They reported that the majority of visitors of the leading smoking cessation website (Quitnet) were female smokers between the ages of 26–44; intended to quit in the next 30 days, and made 5.1 quit attempts during the past year. In a study by Feil *et al*. [39] characteristics of participants and non-participants in an Internet-based diabetes self-management support program were compared. There were no differences found in gender or computer familiarity between participants and non-participants but younger patients and those who had diabetes a fewer number of years were more likely to participate. To our knowledge, no studies examined who participates in website-delivered physical activity promotion programs and who does not.

From a public health perspective it is essential to reach a whole population, as many are at risk. Therefore it is important to study characteristics of participants in online physical activity programs and characteristics of non-participants for whom other dissemination strategies to improve physical activity levels are needed. Special attention should be given on whether a physical activity program reaches those people who are at risk, namely adults with low physically activity levels.

In this paper we examined who participated, and conversely who did not participate in a computer-tailored physical activity intervention delivered through the Internet. The first aim of the current study was to investigate if showing interest (i.e. positive answer on reply card) and actually participation (i.e. completing online assessment at least once) could be predicted by means of individual characteristics such as age, gender, socioeconomic status (SES), employment and physical activity level in particular. The second aim was to report the most common reasons for non-participation.

Based on the literature [23,31-39] we hypothesized that younger people, women, employees, those with higher SES, and those who are already regular physically active would be more likely to show interest and participate in the computer-tailored intervention.

## Methods

### Study sample and procedure

This study was carried out in the context of a randomized control trial that tested the effectiveness of a computer-tailored physical activity intervention program delivered through the Internet. More information about the intervention trial itself and its effectiveness is described in more detail elsewhere [40]. In short, the recruitment for the intervention trial was done as follows: brochures with a call to participate in a physical activity program, with as key element a website-delivered tailored physical activity advice, were distributed indirectly to parents (through their children) of the 14 primary and secondary schools in Belgium, that agreed to participate. People who were interested to receive a tailored physical activity advice through the Internet and fulfilled the inclusion criteria (between 20–55 years old, no history of cardiovascular diseases, access to the Internet at home, work school or public places) could return a reply card mentioning that they wanted to participate. Individuals who did not want to participate were also asked to return the reply card mentioning the reason for refusal. At the start of the intervention trial all individuals who subscribed were invited via e-mail to register on the website, to complete an electronic informed consent statement, and to fill in the diagnostic questionnaire; Participants who were randomized into the intervention groups received their tailored advice on their screen immediately after completing the diagnostic questionnaire; participants in the control waiting group received their tailored advice after they completed the follow-up questionnaire at six months.

For the current study a sub sample of pupils (between 10–18 years of age) from 12 out of 14 intervention schools completed, in the same period in which the brochures were distributed, a short questionnaire in the classroom concerning their parents' age, occupation and physical activity habits. The advantage of this method was that also information could be collected about the parents who did not respond at all to the intervention invitation. Two of the 14 participating schools in the intervention study, refused to let pupils (n = 782) fill in the short questionnaire needed for the current study. The most important reason for refusal was that those schools already participated in a number of studies in the same school year, which caused an excess of questionnaires that had to be completed by their pupils. In the primary schools that participated in the current study, all pupils of 5th and 6th class filled in the questionnaire in the classroom under supervision of their class teacher. In the secondary intervention schools, a number of classes were chosen at random (those that received a study hour in one specific week, caused by sickness of a teacher) to complete the questionnaires in the class room under supervision of an employee of the school. The study was approved by the Ghent University Ethics Committee.

### Measurements

#### Questionnaires completed by the children

The questionnaire existed of two identical parts: one about the mother and one about the father. First, general information (name, age and occupation of mother/father) was asked. Next, questions relating to transportation followed: whether and how mother/father usually goes to her/his work or to the grocery/shops (no transport; car or public transport; by bike; walking). Further, it was asked whether mother/father participates in sports activities, gardening, walking or cycling during leisure time (never; irregular; regular). If appropriate, more details were asked about frequency (days/week) and duration (min/day) of active transportation to work, and about type and frequency (days/week) of sports activities.

A *transport index *(i.e. total min/week of active transportation to work) was calculated by multiplying the frequency and duration of active transportation to work; and a *sports index *was calculated by taking into account the frequency of sport activities of ≥ 3 MET values (multiples of resting metabolic rate, [41]. Further a *total physical activity index *was calculated by counting up the transport index, sports index and all other answers corresponding with participation in a physical activity. Finally, each subject was classified into one of four categories based on these three indices. Category a: a sedentary job (i.e. job-related physical activities of < 3 MET values according to Compendium of Ainsworth, [41]) and almost no active transportation or physical activities in leisure time; category b: irregular or too little physical activities (<150 minutes a week); category c: more then 150 minutes active transportation to work, or regularly participating in physical activities; category d: more then 3 times a week participating in sports activities of ≥ 3 METs. These categories were dichotomized (for binary logistic regression analyses) into a "not regularly active group" (categories a-b; < 150 minutes physical activity a week and < 3 times sport activities a week) and a "regularly active group" (categories c-d; ≥ 150 min physical activity a week or ≥ 3 times sport activities a week).

Parental occupation was used to derive SES by coding the job description according to the classification of the Central Bureau for Statistics of the Netherlands [42]. This classification is based on the level of skills needed to execute a job: low level (e.g. truck drivers); medium level (e.g. nurse); or high level (e.g. dentist). Job descriptions as 'housewife', 'student', 'job-seeker' and 'retired' were classified as 'economically inactive'. Test-retest stability [43] of the questionnaire was evaluated in a different study (data not shown) with 82 pupils between 11 and 13 years of age who completed the questionnaire twice, with a one week interval under supervision of a teacher. The reliability results were good: ICC values of the three physical activity indices ranged from 0.78 to 0.82 for mothers and from 0.70 to 0.82 for fathers; kappa values of the categorical answers all exceeded 0.70 (mothers) and 0.60 (fathers); calculating the proportion of agreement indicated that 91% of the mothers and 80% of the fathers were classified in the same physical activity category, and 97% (mothers) and 91.3% (fathers) were classified in the same SES category.

Other studies showed that children of 11 years and older are able to provide valid data about parent's occupation [44,45] however there are no studies available in which children complete questionnaires concerning their parents' physical activity habits. Based on the study of Hess et al. [46] we hypothesized that the short questionnaire used in the current study may have underreported the actual behavior as it was completed by an intermediary.

#### Reply cards of parents

Information collected during the recruitment process of the intervention study [40] was also used in the current study. Basic information was collected from all parents that responded (n = 1998) to the invitation by means of a reply card in the brochure: name and family name of the adult; relation to the child; and name and grade of their child(ren). Further, individuals who were interested in the intervention trial filled in contact information and those who did not want to participate mentioned the reason for refusal (not fulfilling the inclusion criteria; already sufficiently physically active; no interest; other reason).

### Statistical analyses

Chi^2 ^analysis was used to determine possible differences in physical activity level (not regularly active versus regularly active) between parents who showed interest, participated or did not participate.

Binary logistic regression was used to detect if showing interest into the intervention could be predicted by age, gender, SES, employment or baseline physical activity level. As there were lots of good intenders but few completers, binary logistic regression was also done to detect which variables could predict *participation*.

All statistical analyses were performed using SPSS software (version 12.0) and statistical significance was set at a level of 0.05.

## Results

### Showing interest to and participate in the intervention

In total 1998 parents returned the reply card of the brochure, mentioning whether they wanted to participate in the study or not. Most of the responders were mothers (60.6%).

Of the 2000 short questionnaires that were distributed in the intervention schools, 1740 (87%) were completed by pupils. 1730 questionnaires were suitable for further analysis and were checked on doubles. Eighty-one siblings, reporting on the characteristics of the same couple of parents, were detected and only one questionnaire of them was (at random) included in the analysis. This resulted in 1649 questionnaires of which characteristics of 3232 parents were computed (1637 mothers and 1595 fathers).

**Figure 1:**
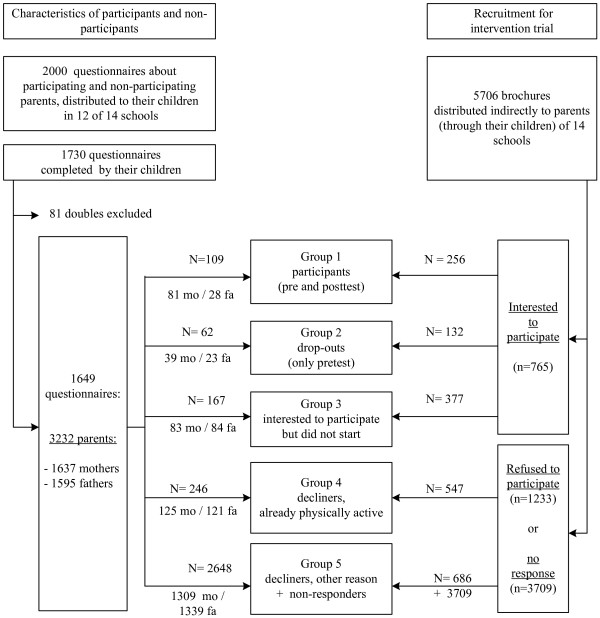
Schematic overview of the study sample. Study groups based on whether parents showed their interest in the intervention, whether they completed baseline and follow-up assessment and on the reason for non-participation. Note: mo = mothers; fa = fathers.

The 3232 names on the questionnaires filled in by the pupils were matched with the names of the participants in the intervention trial and with those who did not participate but filled in the reply card (showing their interest for the intervention or reason for refusal). Based on whether parents completed baseline and follow-up assessment and on the reason for non-participation, five groups of parents were identified, and were subject to further analyses in the current study. Group 1 consists of the parents who logged in to the website and completed the diagnostic questionnaire both at baseline and at follow-up (n = 109); subjects in group 2 are the parents who logged in to the website and completed the diagnostic questionnaire at baseline but did not return to the website at follow-up (n = 62); group 3 contains the responders who showed there interest in the intervention but did not start (did not visit the website to complete diagnostic questionnaire at baseline) (n = 167); group 4 consists of the parents who did not want to participate in the intervention because they were already sufficiently physically active (n = 246); and group 5 includes the remaining individuals (n = 2648): they declined for other reasons or did not respond to the intervention invitation. See figure 1 for an overview of the study sample.

For binary logistic regression analyses these five groups were dichotomized into "participants" (groups 1–2) and "non-participants" (groups 3–5) and also into "interested parents" (groups 1–3) and "not-interested parents" (groups 4–5).

### Physical activity levels

Table 1 shows the parents' physical activity levels, reported by their children, in the five groups (Table 1). Only a small proportion of them (25%) met the physical activity recommendations for adults (at least 30 minutes of moderate-intensity physical activity on most, preferably all days of the week; or participating in at least 20 minutes of continuous vigorous-intensity physical activity 3 times a week [12,47]) and Chi^2 ^analysis showed that this proportion significantly differed between groups (χ^2 ^= 17.02, p < 0.01). Subgroup analyses focusing on who were interested in the intervention (groups 1–3) and those who did not participate in the intervention (groups 3–5) showed different results for mothers and fathers. Mothers in group 3 are tended to be less physically active then mothers in group 1 and 2; no significant differences were found between those groups who did not participate. On the contrary fathers in group 4 are the most active of the non-participants; and no significant differences were found between fathers in the three groups who were interested to participate in the study (Table 1).

**Table 1:** Children-reported physical activity characteristics of their parents in both participant and non-participants groups

	**Compliance with physical activity recommendations**^**a**^					
	
	**Mothers**		**Fathers**		**Total sample**	
	
	**N**	**%**	**N**	**%**	**N**	**%**
**Group 1**	26/81	32.1	5/28	17.9	31/109	28.4
**Group 2**	12/39	30.8	7/23	30.4	19/62	30.6
**Group 3**	17/83	20.5	25/84	29.8	42/167	25.1
**Group 4**	35/125	28.0	52/121	43.0	87/246	35.4
**Group 5**	291/1309	22.2	345/1339	25.8	636/2648	24.0
**Total**	381/1637	23.3	434/1595	27.2	815/3232	25.2
	
	**X^**2**^**	***P***	**X^**2**^**	***P***	**X^**2**^**	***P***

**groups 1–5 **(all)	7.48	0.11	18.23	0.00**	17.02	0.00**
**groups 1–3 **(interested)	3.11	0.05^(*)^	0.62	0.28	0.71	0.24
**groups 3–5 **(not participated)	2.40	0.30	16.81	0.00***	15.46	0.00***

Note: Group 1 = participated both at baseline and follow-up; Group 2 = participated at baseline and dropped-out; Group 3 = showed interest in the intervention, but did not start; Group 4 = declined, because they find themselves already physically active enough; Group 5 = declined for other reasons or did never respond

^a ^= accumulating at least 30 minutes of moderate-intensity physical activity on 5 days of the week OR doing at least 20 minutes of continuous activity at vigorous intensity, at least three times a week.

^(*) ^P < 0.1

** P < 0.01

*** P < 0.001

### Predicting interest and participation

Results of binary logistic regression analysis predicting parents' interest and participation are shown in table 2.

**Table 2:** Results of binary logistic regression predicting parent's interest (i.e. showed their interest in intervention by positive response on reply card) and participation (i.e. completed online assessment) in a physical activity intervention delivered through the Internet

**MODEL 1**	**Showing interest **^**a **^**(all groups, n = 2637)**					**participation **^**b **^**(interested, n = 300)**				
		
	**n**	**n interested (%)**	**adjusted OR**	**95% CI**	***P***	**n**	**n participated (%)**	**adjusted OR**	**95% CI**	***P***
	
Gender										
male	1427	128 (9.0)	1.00^c^			128	48 (37.5)	1.00^c^		
female	1210	172 (14.2)	1.68	1.31–2.16	**<0.001**	172	101 (58.7)	2.27	1.39–3.70	**0.001**
SES										
low	879	47 (5.3)	1.00^c^			47	14 (29.8)	1.00^c^		
medium	1489	213 (14.3)	2.87	2.07–3.99	**<0.001**	213	121 (56.8)	3.17	1.58–6.34	**0.001**
high	269	40 (14.9)	3.42	2.18–5.37	**<0.001**	40	14 (35.0)	1.65	0.65–4.19	0.293
PA										
not-active	1908	216 (11.3)	1.00^c^			216	106 (49.1)	1.00^c^		
active	729	84 (11.5)	0.93	0.71–1.22	0.584	84	43 (51.2)	0.92	0.54–1.57	0.766
**MODEL 2**	**Showing interest **^**a **^**(all groups, n = 2976)**					**participation **^**b **^**(interested, n = 317)**				
		
	**n**	**n interested (%)**	**adjusted OR**	**95% CI**	***P***	**n**	**n participated (%)**	**adjusted OR**	**95% CI**	***P***
	
Gender										
male	1452	128 (8.8)	1.00^c^			128	48 (37.5)	1.00^c^		
female	1524	189 (12.4)	1.70	1.34–2.17	**<0.001**	189	112 (59.3)	2.38	1.49–3.80	**<0.001**
Employed										
no	339	17 (5.0)	1.00^c^			17	11 (64.7)	1.00^c^	0.53–1.46	0.626
yes	2637	300 (11.4)	3.03	1.81–5.06	**<0.001**	300	149 (49.7)	0.88		
PA										
not-active	2204	229 (10.4)	1.00^c^			229	114 (49.8)	1.00^c^		
active	772	88 (11.4)	0.94	0.72–1.22	0.630	88	46 (52.3)	0.77	0.27–2.19	0.619

Note:

^a ^including group 1, 2, 3, 4, 5

^b ^excluding group 4&5

^c ^Reference group

CI = confidence interval; PA = Physical activity; SES = Socio-economic status (based on occupation); adjusted OR = odds ratio adjusted for gender, SES/employment and physical activity; CI = confidence interval; PA = Physical activity

Two models were tested: In the first model 'gender' (male-female), 'SES' (low-medium-high) and 'physical activity level' (not active-active) were included as predictors; in the second model, 'SES' was replaced by 'employed' (no-yes) to include also the economically inactive parents (n = 339). Age was not included in the models as it was not a predictor of interest or participation. In 256 out of 3232 questionnaires the job occupation of the parent was missing or to vague to code the job. This resulted in 2637 parents that were included in model 1 and 2976 parents in model 2 (Table 2).

Gender could predict both interest and participation in the intervention trial in both models: mothers were consistently more likely to show interest and participate in the intervention trial then fathers. SES was also a significant predictor: parents of medium or high SES were more likely to show interest in the study than parents of low SES. However when we only take into account the parents who agreed to participate (group 1, 2 and 3), those in the medium SES group were more likely to complete baseline questionnaires compared to persons in the low and high SES groups. In the second model, 'employed' predicted having interest (those with a job were more likely to show interest then those without a job) but when persons were interested, this variable could no longer predict further participation. Finally, baseline physical activity level did not predict interest nor participation in any of the models (Table 2).

### Reasons for non-participation

Reasons for refusal are shown in table 3. The most important reasons were: "do already a lot of physically activities" (44%) and "having no interest" (36%). Almost nobody (2%) mentioned "not having PC or Internet access" as a reason for not participating in the study (Table 3).

**Table 3:** Reasons given by parents (N = 1233 of 5706) for refusal to participate in a physical activity intervention delivered through the Internet.

	**N**	**Percentage**
Already physically active	543	44
No interest	448	36
Not eligable	47	4
No PC or Internet access	26	2
Not specified	114	9
Others:		
No time or too busy	40	3.2
Already showed their interest in the intervention via other child	8	0.7
Health concerns	6	0.5
Not specified	1	0.1

## Discussion

This study is one of the few that compared participants and non-participants of an interactive computer program delivered through the Internet and the first to compare participants and non-participants, recruited through schools, of a website-delivered physical activity intervention. Interventions to promote physical activity should reach a wide variety of people at once, at any time and location. This study reported that young and older as well as physically inactive and physically active adults could be reached by an interactive computer-tailored physical activity intervention when brochures were distributed in schools. However, more women then men and more adults of medium SES then adults of low SES participated. Further more employees than economically inactive people were interested in the intervention program. Most reported reasons for non-participation were (1) being already sufficiently physical active and (2) not interested.

A main finding of the study is that there are important gender differences in study interest and participation: mothers were more likely to show interest and to participate then fathers. Other studies also found a greater proportion of women participating in website-delivered health promoting programs [33-36], but the results for participation in physical activity promoting programs are mixed [29,48]. The latter is in accordance with the finding that despite the fact that women are more likely to search for health information through the Internet than men, both women and men are equally interested in online information related to exercise and fitness [49]. However, it has to be mentioned that, similar with the current study, most website-delivered intervention studies recruited their participants by advertisements and did not recruit proactive (i.e. 'directly contacting potential participants and offering services to them' [50]). Therefore it is not sure that in the mentioned studies an equally proportion of men and woman have been reached. In our study it is possible that more women received and read the brochure with the invitation to receive a tailored advice, because mothers are often more involved in child care (for example: 285 (18%) children reported in the questionnaire that their mother was a "housewife", in contrast with only 7 (0.4%) children who described their fathers occupation as "househusband").

A second finding is that employed parents and those with higher level of SES, were more likely to show interest than non-employed parents or parents classified in the low SES group. This is similar with the results of non-Internet based studies [31,51] who consistently found lower rates of participation in work-site wellness programs in low SES groups than those in higher SES groups. However, in the current study only medium SES could predict both interest and participation, while employment and high SES could only predict interest. It seems that interested people who are economically inactive, are motivated enough to visit the intervention website and complete the assessment questionnaires. Further, it seems that people of higher SES (e.g. a bank manager) are indeed interested in the intervention material, but their busy time schedule (inherent to their profession) prevents them to actually participate.

In general, it is stated that participants in health promoting programs, have already more positive health behaviors (for example being regularly physically active) compared with the general population [31,51]. However, in our study no differences were found between regular physically active and non-physically active parents in predicting interest or participation: mothers or fathers who were regular physically active were not more likely to show interest or to participate in the intervention trial then irregular physically active parents. However, the physical activity level of mothers differed between those who agreed to participate and those who actually participated: mothers who showed interest but did not participate were less regularly active compared with those who did participate (completed pre or posttest questionnaires). This was not the case for the fathers.

It is important to note some limitations and strengths of this study. The first limitation is that parent's characteristics such as occupation and physical activity level were reported by the children and not by the parents themselves. However, studies showed that children of 11 years and older are able to provide valid data about parents' occupation in a survey setting [44,45] and test-retest stability of the questionnaire used in the current study showed good results. Although, there are important benefits to let children report about their parents' physical activity behavior, such as collecting data from precontemplators (which is difficult to get as they are not willing to complete physical activity assessments), there is still a possibility that this short questionnaire filled in by an intermediary underreport the actual parents' physical activity behavior [46].

A second limitation of the study is the indirect recruitment method that was used by distributing brochures to parents through their children. It is possible that this method induced a coverage error: As already mentioned we could not know how many pupils handed out the brochure to their parents and whether both parents read it. As a consequence no response and participation rates could be measured exactly. However, the used recruitment method also had several advantages.

The first strength is that by recruiting participants via an offline method, we could also reach irregular Internet users. Second, by using the school as setting to recruit we could offer parents without Internet access a computer session on the school site. However, similar with the literature [52] we found that Internet access in this population is very high: no computer sessions were organised by lack of interest and almost nobody mentioned lack of Internet access as reason for not-participating. Third, using the school setting also created the possibility of collecting data of non-participants via their children and fourth, the way the intervention program was offered to the participants mimicked a real-life implementation as good as possible.

## Conclusion

It seems that the evaluated computer-tailored physical activity intervention delivered through the Internet did not reach only the most actives or the younger adults. The results showed that both physically active parents and sedentary parents were interested and participated; and that younger and older adults were equally likely to participate. However, the program did not succeed in reaching all underserved populations, for example those parents of low SES. Other health-education programs and/or other recruiting methods are needed to reach all segments of the adult population, especially those high at risk.

More research is required on how to reach these population subgroups (inactive men, adults of low SES) that are less likely to participate in our website-delivered intervention. Could they be reached by another strategy (for example by adding a face-to-face contact during recruitment [53]) or would it be more cost-effective to engage subgroup participants in other kinds of interventions?

## Competing interests

The author(s) declare that they have no competing interests.

## Authors' contributions

HS participated in the design of the study, collected and analyzed the data, and led the writing of the paper. ID participated in the design of the study and provided substantive feedback on the manuscript. Both authors have read and approved the final manuscript.
